# Genome-Wide Association Study and Post-genome-Wide Association Study Analysis for Spike Fertility and Yield Related Traits in Bread Wheat

**DOI:** 10.3389/fpls.2021.820761

**Published:** 2022-02-11

**Authors:** S. Sheoran, S. Jaiswal, N. Raghav, R. Sharma, Sabhyata, A. Gaur, J. Jaisri, Gitanjali Tandon, S. Singh, P. Sharma, R. Singh, M. A. Iquebal, U. B. Angadi, A. Gupta, G. Singh, G. P. Singh, A. Rai, D. Kumar, R. Tiwari

**Affiliations:** ^1^Indian Council of Agricultural Research-Indian Institute of Wheat and Barley Research, Karnal, India; ^2^Indian Council of Agricultural Research-Indian Agricultural Statistics Research Institute, New Delhi, India

**Keywords:** GWAS, 35K, MTAs, spike fertility, yield, wheat

## Abstract

Spike fertility and associated traits are key factors in deciding the grain yield potential of wheat. Genome-wide association study (GWAS) interwoven with advanced post-GWAS analysis such as a genotype-phenotype network (geno-pheno network) for spike fertility, grain yield, and associated traits allow to identify of novel genomic regions and represents attractive targets for future marker-assisted wheat improvement programs. In this study, GWAS was performed on 200 diverse wheat genotypes using Breeders’ 35K Axiom array that led to the identification of 255 significant marker-trait associations (MTAs) (*–log_10_P* ≥ 3) for 15 metric traits phenotyped over three consecutive years. MTAs detected on chromosomes 3A, 3D, 5B, and 6A were most promising for spike fertility, grain yield, and associated traits. Furthermore, the geno-pheno network prioritised 11 significant MTAs that can be utilised as a minimal marker system for improving spike fertility and yield traits. In total, 119 MTAs were linked to 81 candidate genes encoding different types of functional proteins involved in various key pathways that affect the studied traits either way. Twenty-two novel loci were identified in present GWAS, twelve of which overlapped by candidate genes. These results were further validated by the gene expression analysis, Knetminer, and protein modelling. MTAs identified from this study hold promise for improving yield and related traits in wheat for continued genetic gain and in rapidly evolving artificial intelligence (AI) tools to apply in the breeding program.

## Introduction

Wheat is an important cereal providing 20% of calories and protein for the human diet globally (Shiferaw et al., 2013). With the predicted global population of approximately 9 billion in 2050, the demand for wheat is expected to increase by close to 70% (Su et al., 2016). However, in contrast to the required growth rate of 2.4% increase in grain yield per year, we are having merely a rate of.9% and at this rate, we can achieve only a 38% increase in the present yield by 2050 (Ray et al., 2013). Moreover, adverse impacts of climate change, diminishing natural resources, rapidly evolving new threats of pests and pathotypes, and genetic erosion would further add obstacles to the achievement of doubling the yield potential in the stipulated time. Thus, there is a clear need to improve our understanding of the genetic architecture of grain yield in our working gene pool and to search for the efficient utilisation of available genetic resources.

Grain yield has a complex underlying genetic architecture that depends on several related traits. Furthermore, high genotype x environment (GxE) interaction and low heritability of this ultimate trait in most cases, make the selection process most challenging. In such a situation, indirect selection of grain yield *via* highly heritable (*h*^2^) correlated traits would be practically more feasible. One of the key contributing traits for keeping the high yields are spike-related traits such as spike fertility.

Spike fertility is an index defined by the number of grains produced over the chaff weight which can be attributed to siphoning assimilates to the grains in the florets than to the other parts of the spike. Acreche et al. (2008) reported an increase in grain number was related to improvement in both spike weight and fruiting efficiency (SF). This becomes important in the light of the observation that an increase in the number of fertile florets per gram of spike was not at the cost of a reduction in the grain weight in improved cultivars. It is also well known that the number of grains per meter square directly correlates with yield enhancement (Acreche et al., 2008). The effect of various agronomic and physiological traits on grain yield was recently reviewed by Tshikunde et al. (2019). These traits affect grain yield through photosynthetic efficiency, input use efficiency, grain filling rate, and dry matter translocation (Li et al., 2019). Extensive use of limited genetic resources with semi-dwarf stature and wheat-rye translocations (e.g., 1BL.1RS), in global wheat breeding programs, have caused a near fixation of these genes as well as significant genetic erosion (Girma, 2017; Würschum et al., 2017). This indicates the need of improving our knowledge on the genetic architecture of grain yield and other attributing traits jointly with the exploration of causal variants in genetic resources for their efficient utilisation in wheat grain yield improvement programs.

High throughput genotyping using NGS-derived markers such as single nucleotide polymorphisms (SNPs) leads to dense and uniform coverage of all the chromosomes receiving impetus. The future genetic gain is more likely from a genomics-driven breeding program which requires an in-depth understanding of all the major/minor quantitative trait loci (QTLs) segregating in the elite germplasm pools. GWAS has been found a powerful tool for dissecting complex traits by finding causative allelic variation at individual SNP markers or associated with natural phenotypic variation (Alqudah et al., 2020) which can be used effectively to fine map these traits (Garcia et al., 2019; Sheoran et al., 2019; Alqudah et al., 2020; Kumar et al., 2020; O’Connor et al., 2020; Sehgal et al., 2020). This approach has been widely used to predict phenotypically related candidate genes in many crops (Si et al., 2016; Wang et al., 2016; Liu et al., 2018). GWAS revealed common QTLs between floret fertility, spike morphology, assimilate partitioning efficiency, and yield, suggesting genetic association controlling these complex traits (Guo et al., 2017). It overcomes the limitation of bi-parental mapping by using a population of unrelated diverse genotypes representing all possible recombination events. The widespread availability of cost-effective genotyping techniques such as genotyping by sequencing (GBS) and SNP arrays (35K Breeder array, 90K iSelect gene chip, *etc*.) have further improved the resolution of GWAS as well as accuracy and predictability of candidate genes and QTL regions while accounting the causal variants in the population. Now, the reference genomes of hexaploid wheat (*Triticum aestivum* L., International Wheat Genome Sequencing Consortium [IWGSC], 2018), wheat A sub-genome (*T. urartu*, Ling et al., 2018), and D-subgenome (*Aegilops tauschii*, Luo et al., 2017) have been made available which facilitates the fine mapping, gene discovery and cloning in wheat (Pang et al., 2020; Tura et al., 2020).

Keeping this in mind, GWAS was performed using Axiom Wheat Breeder 35K Genotyping Array on a panel of 200 wheat genotypes consisting of indigenous collections, elite lines, released varieties, genetic stocks, and exotic lines. Genotypes were characterised for spike fertility, yield, and related traits for three consecutive years. The objectives of the study were (i) to identify novel MTAs linked to grain yield and yield-related traits, (ii) to identify candidate genes of these MTAs and to investigate their underlying functions, and (iii) to construct a genotype-phenotype network (geno-pheno network) for yield improvement to further narrow down the promising SNPs associated with trait (Pradhan et al., 2019).

## Materials and Methods

The experiment was conducted at the ICAR-Indian Institute of Wheat and Barley Research (IIWBR), Karnal (29^0^ 42′ N, 77^0^ 2′ E) over three consecutive years *viz*., 2016–2017, 2017–2018, and 2018–2019 using the recommended agronomic practices. An alpha-lattice design with three replications was followed where the planting was done in plots using a handheld IIWBR dibbler (Sharma et al., 2016) in four rows each. A subset of 200 diverse bread wheat (*T. aestivum* L.) genotypes was chosen for the present GWAS (Supplementary Table 1) from the plant material used by Sheoran et al. (2019). Each year seeds were planted under timely sown condition (1st week of November) in a plot size of 1m^2^. Each genotype occupied a single plot of dimension 1.25 m ×0.8 m. In each locus, two seeds were planted and after 15 days of sowing, one plant was maintained per locus hence 48 plants were finally retained in each plot. Each plot had four rows with 12 plants per row. Row to row spacing was 20 cm and plant to plant spacing was 10 cm with seedling depth at 5 cm.

### Phenotyping

In the phenotyping experiment, 200 genotypes were evaluated for 15 agro-morphological traits including some less explored traits *viz.*, days to heading (DH), days to anthesis (DA), days to maturity (DM) were recorded as number of days taken from sowing to the days when 75% plants shown spike emergence, anthers emergence, browning of spikes respectively. Plant height (PH) was measured from the base of the plant to the tip of the spike (excluding awns), and spike length (SL) was measured from the tip of the apical spikelet (excluding awns) to the base of the spike at the time of physiological maturity. Other considered traits were spike dry weight (SDW), grain number per spike (GNS), grain weight per spike (GWS), thousand-grain weight (TGW), grain yield (GY), spike chaff weight (CW), and spike fertility (SF). Tillers per plant (TP) were calculated as the largest number of tillers produced by a plant. Biomass (BM) was the total weight of plants per plot and harvest index (HI) was calculated as (GY/BM)*100. All the observations were recorded on the main tillers of nine plants per plot randomly tagged at the booting stage, except for TGW, GY, and BM that were recorded on a per plot basis.

### Statistical Analysis

ANOVA and estimates of repeatability were calculated using the mixed procedure in SAS 9.3 software, 2011^1^. Best linear unbiased predictions (BLUP) were made for each genotype for each trait by combining data from three environments using mixed linear models (MLM) fitted with restricted maximum likelihood (REML) methods where the effect of blocks is considered random. Heritability for each trait over the environments was estimated using META-R (Alvarado et al., 2020). The phenotypic data were later divided into four datasets each coming from three environments *viz*., E1 (2016-17), E2 (2017-18), E3 (2018-19), and BLUP. The R software^2^ was used to calculate descriptive statistics and summarisation of data for each data set using command *summary()* and ggplot2 package, respectively whereas, the correlation matrix among BLUP values of studied traits was calculated using command *cor()* and visualised with ‘corrplot’ package.

### Single Nucleotide Polymorphisms Calling and Linkage Disequilibrium

Genotypic data with 35K Axiom Wheat Breeders Array were obtained from the IIWBR database, details of which are mentioned in Sheoran et al. (2019). Markers with minor allele frequency (MAF) of less than 5%, more than 10% missing values, and individuals with more than 15% missing SNP calls were removed from the dataset. Markers with no chromosomal position based on a high-density consensus map generated by using the mapping population (Allen et al., 2017) were also removed. For association analysis, three environments, namely E1 (2016–2017), E2 (2017–2018), E3 (2018–2019) in three replications and BLUP were considered. Linkage disequilibrium (LD) analysis was performed across A, B, and D genomes. Intra-chromosomal pairwise marker LD as squared allele-frequency correlations (*r*^2^) values were calculated in TASSEL v5.2 (Bradbury et al., 2007) using a sliding window approach with default parameters. As a function of genetic distance, the estimated *r*^2^ values for significant SNP marker pairs were plotted to understand the extent of LD. A second-degree “loess” function (Cleveland, 1981) in the R statistical program was fitted to estimate the rate of LD decay over genetic distance (cM).

### Population Structure

Population structure analysis was performed on 15,178 markers from 200 genotypes. The input file was prepared using the Perl script. Parallelisation of STRUCTURE 2.3.1 (Chhatre and Emerson, 2017) run was done based on command line in-house C++ MPI programming in Linux reducing the computation load many-folds. The parameters used for running were 100,000 iterations of burn-in and 100,000 Monte Carlo Markov Chain (MCMC) iterations. *K* values tested were from 2 to 10 with five iterations run for each *K*. Number of subpopulations (*K*) that are more likely, i.e., ΔK statistics which relies on the rate of change in log probability [LnP(D)] between successive K values were analysed using STRUCTURE HARVESTER (Earl and vonHoldt, 2012). Based on the best *K* value bar plot and fixation index (*F*_*st*_) of each sub-population was generated using STRUCTURE run.

### Genome-Wide Association Study and Genotype-Phenotype Network

Association analysis was performed using a compressed mixed linear model (CMLM) by the GAPIT package, which takes into account the results of population stratification and kinship as a covariate to minimize false positives (Lipka et al., 2012). GWAS analysis was conducted between SNP markers and phenotypic data in individual environments and BLUP values across all environments. A threshold *P*-value of.001 (*–log_10_P* = 3) was used to declare significant SNPs for GWAS results. VanRaden kinship (K) matrix (VanRaden, 2008) for the 200 genotypes was also generated using GAPIT.

To identify the key SNPs associated with one or more traits, genotype-phenotype network analysis has been carried out using the Network-Based Genome-Wide Association Studies in (*netgwas*) R package (Behrouz et al., 2017). netphenogeno reconstructs the conditional dependence network among genetic markers, phenotypes, and between genetic markers and phenotypes. The intra- and inter-chromosomal conditional interactions among genetic loci were also calculated using the ‘*netsnp’* of the ‘*netgwas*’ package.

### Candidate Gene Prediction and Homology Modeling

Genes associated with the stable loci detected in this study were identified using the EnsemblPlants database^3^ and the International Wheat Genome Sequencing Consortium (IWGSC)^4^ *RefSeq v1.1* annotations. Nearby regions (1.5 kb upstream and downstream) of stable MTA were also selected to find out candidate genes. Expression analysis has been done using the RNA-seq expression database of polyploid wheat^5^ which includes RNA-seq datasets of multiple tissues and developmental time courses. Expression values are represented in Fragments per kilobase of transcript per million mapped reads (FPKM).

Homology modeling was carried out to deduce the proteins translated by candidate genes of selected SNPs. For this purpose, query sequences were subjected to position-specific iterative (PSI) BLAST against protein data bank database (PDB)^6^. The top three templates showing minimum *E*-value and highest similarity percentage were selected for 3D structure prediction in the SwissModel server^7^. Values from ERRAT, Verify3D, Ramachandran plots, and FATCAT tools were used to predict and validate the best 3D structure.

## Results

### Phenotypic Analysis and Population Structure

**FIGURE 1:**
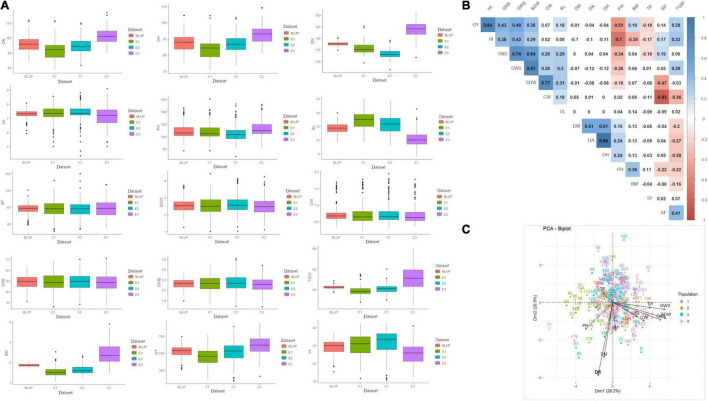
**(A)** Summary of data observed for 15 agro-phenological traits, **(B)** correlation among studied traits, and **(C)** genotype-trait biplot summarizing the genetic variability. In figure, BLUP, Best Linear Unbiased Prediction; E1, the year 2016–2017; E2, the year 2017–2018; E3, the year 2018–2019; DH, days to heading; DA, days to anthesis; DM, days to maturity; PH, plant height; SF, spike fertility; CW, chaff weight; SDW, spike dry weight; SL, spike length; GNS, grain number per spike; GWS, grain weight per spike; TGW, thousand grains weight; GY, grain yield; TP, tiller per plant; HI, harvest index; BM, biomass.

The phenotypic performance of 200 genotypes based on investigated traits in three environments and BLUP is summarised in Figure 1A and Supplementary Table 2. The coefficients of variation (CV) for these 15 traits ranged from 1.6 to 43.8%, showing broad phenotypic variation and considerable improvement potential. Substantial phenotypic variations among genotypes and datasets were reported for all the studied traits excluding SL, CW, SDW, GWS, GNS, and BM for which the mean sum of squares (MSS) for the environments and BLUP was non-significant at *P <* 0.001 (Supplementary Table 3). GxE interaction was also significant (at *P <* 0.001) for studied traits except for TP. Heritability of all 15 traits ranged from 0.3 (BM) to 0.97 (DH), indicating that both genetic and environmental factors played important role in the phenotypic expression of these measured traits (Supplementary Table 3). Besides, broad-sense heritability estimates were found highest for CW (0.92) followed by SDW (0.87) and GNS (0.86). Furthermore, the genotype-trait biplot indicated sufficient contribution of different genotypes from various sub-populations to the diversity of studied traits (Figure 1C). Pearson’s correlation coefficients, based on BLUP values of fifteen agronomical traits, ranged from –0.83 (CW vs. SF) to 0.99 (DH vs. DA) at *P <* 0.05 (Figure 1B). GY showed a positive correlation with GWS, GNS, SDW, SL, BM, TGW, and HI which ranged between 0.18 (SL) to 0.49 (GWS) besides HI (0.84). SF showed a significantly positive correlation with HI and GNS whereas, a significant but negative correlation with SDW, CW, and PH. The correlation between SF and GY was positive but non-significant (Figure 1B).

### Genomic Coverage, Population Structure, and Linkage Disequilibrium

**FIGURE 2:**
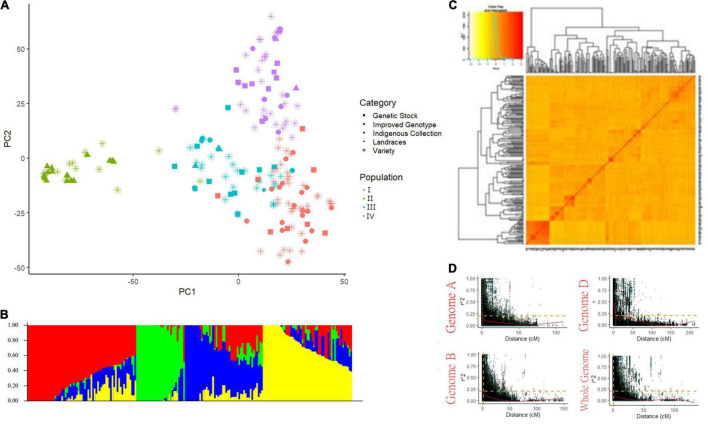
Population structure of genome-wide association study (GWAS) panel consisted of 200 bread wheat genotypes **(A)** PCA plot, **(B)** Bar plot showing the number of optimum sub-population in GWAS panel, **(C)** Van Raden kinship matrix, and **(D)** Scatter plot showing LD decay in three sub-genomes and whole genome.

A total of 15,178 SNP markers covering 4529.51 cM map distance with an average distance of 0.5 cM were found polymorphic after filtering data according to Sheoran et al. (2019). No genotype was removed for low genotypic data (MIND > 0.01). Among polymorphic markers, 39.45, 50.2, and 10.2% were reported on the A, B, and D genomes, respectively (Supplementary Table 4). Chromosome 2B had the highest number of markers (1412) while the 4D chromosome spanned the lowest number of markers (58). The average genetic diversity (GD) and the polymorphism information content (PIC) for the whole genome were found at 0.35 and 0.28, respectively. The average GD and PIC across the genome were observed highest for the B genome (0.36 and 0.29) and lowest for the D genome (0.34 and 0.27) (Supplementary Table 4). Population STRUCTURE analysis stratified the present GWAS panel into four optimum sub-populations comprising 67, 30, 48, and 55 genotypes respectively falling in subgroups I, II, III, and IV with admixture (Figures 2A–C). The first subgroup (I) has predominantly released varieties and improved genotypes (∼80%), mostly post-green revolution high-yielding varieties with complex pedigree. Subgroup II consists of the indigenous collection and tall traditional type genotypes possessing tolerance to heat and drought conditions. Subgroup III has major components as genotypes suited for hills, warmer areas, and disease-resistant lines while subgroup IV has genotypes adapted to varying environments. Principal component and kinship analyses also showed four groups, which corresponded to the four sub-populations revealed by Structure (Figures 2A–C). LD was estimated by calculating the squared allele frequency correlation (*r*^2^) among all possible pairs of markers for each of the 21 chromosomes. Obtained *r*^2^ values were then plotted against genetic distance (cM) for each of the three genomes separately and across the whole genome (Figure 2D). LD decayed at 1, 1.3, and 5.8 cM for A, B, and D genomes, respectively at cut-off *r*^2^ = 0.2, while for the whole genome, decay was observed at 1.3 cM. Based on this average LD decay, the size of QTL was estimated, i.e., all significant SNPs within 1.3 cM were considered as part of the same QTL.

### Significant Loci Associated With the Traits

**FIGURE 3:**
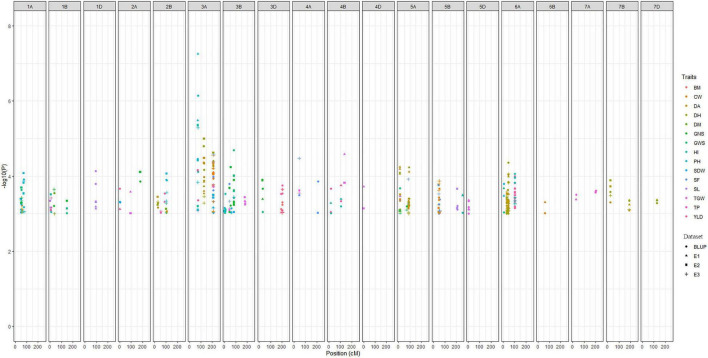
Manhattan plot summarizing the significant MTAs reported for fifteen traits in four datasets. In figure, BLUP, Best Linear Unbiased Prediction; E1, the year 2016–2017; E2, the year 2017–2018; E3, the year 2018–2019; DH, days to heading; DA, days to anthesis; DM, days to maturity; PH, plant height; SF, spike fertility; CW, chaff weight; SDW, spike dry weight; SL, spike length; GNS, grain number per spike; GWS, grain weight per spike; TGW, thousand grains weight; GY, grain yield; TP, tiller per plant; HI, harvest index; BM, biomass.

The main objective of this study was to identify the major MTAs for the traits associated to improve grain yield and adaptability in wheat. Accordingly, for all 15 traits, a total of 255 significant MTAs were identified across three environments (E1, E2, and E3) and BLUP in the present study (Figure 3). False discovery rate (FDR) was significantly controlled with MLM statistics as seen in Q-Q plots (Supplementary Figure 1). A maximum of 52 MTAs was reported on chromosome 6A for eight traits *viz*., DA, DH, PH, SDW, GWS, GY, BM, and HI whereas, only one MTA each was reported on chromosome 4D and 6B for TGW and CW, respectively. The distribution of MTAs on three subgenomes was 154 (A), 75 (B), and 25 (D). Significant loci from chromosome 1D and 6B from this study were exclusively associated with SL and CW, respectively. Likewise, loci reported on chromosomes 4D and 5D were associated with TGW. All the MTAs consistently associated with the trait in at least one environment and BLUP were considered stable loci for the respective trait. Among traits, a maximum of 36 MTAs was detected for DH and a minimum of 6 for BM. Phenotypic variations explained by these traits ranged from 5% (DM) to 45% (PH). The highest *–log_10_P* value 7.25 was reported for the marker AX-94407346 (3A: 74.06 cM) associated with PH having a negative allelic effect of –12.69. Detailed results on MTAs are given in Supplementary Table 5.

### Phenological Traits

A total of 98 loci were found associated with four phenological traits (DH, DA, DM, and PH) (Supplementary Table 5). For DH, 36 significant MTAs were detected on chromosomes 1A (1), 2B (4), 3A (3), 5A (6), 6A (19), 7B (2), and 7D (1). Three MTAs (AX-94712794, AX-94805904, AX-94842717) on chromosome 3A and two MTAs (AX-94724484, AX-95136668) on chromosome 6A for DH were found stable across the three environments and BLUP explaining phenotypic variation ranging from 26.2 to 33.4%. For DA, 26 significant MTAs were observed on chromosomes 1A (1), 2B (4), 3A (3), 5A (6), 6A (10), and 7B (2) accounting for 23.0–33.0%. However, two stable and consistent MTAs were found on chromosome 3A (AX-94842717, 209.17 cM) and chromosome 6A (AX-94724484, 47.56 cM) for DA (Supplementary Table 5). Out of 16 MTAs associated with DM, only 2 MTAs on chromosome 1B (AX-95161998) and 2B (AX-94853276) were observed in an environment and BLUP explaining 5–10.2% of the phenotypic variations. A total of 20 MTAs for PH on chromosomes 1A (14), 2B (1), 3A(2), 3B (2), and 6A (1) were detected. Interestingly, 14 SNPs were mapped on chromosome 1A within the map position of 74.11–74.86 cM that collectively explained 43% of phenotypic variation for PH. Another most stable region for PH was observed on chromosome 3A at an interval of 0.75 cM and was found consistent explaining phenotypic variation ranging from 36.8 to 45.4%.

### Yield Contributing Traits

For TP, 21 loci were reported on chromosomes 1A (16), 2B (1), 5A (1), 7A (2), and 7B (1). Two MTAs, AX-94757176 (1A) and AX-94446620 (7A) were consistent in E2 and BLUP whereas, the rest was reported only with BLUP values (Supplementary Table 5). These SNPs accounted for 8–11% variation for TP. All the markers reported on chromosome 1A and 7A belong to the same genetic position 74.11 and 201.13 cM, respectively. For SL, 10 loci accounting for the phenotypic variation of range between 7 and 28% were found on chromosomes 1B (2), 1D (3), 3B (1), 5B (2), and 7A (2). Marker AX-94629635 (95.7 cM) on chromosome 1D was found to be most stable for SL among all due to its consistency over two environments (E2, E3) and BLUP with a negative allelic effect (–0.48).

For GWS, a total of 15 significant MTAs were identified on chromosome 1A (5), 3B (3), and 6A (2), and one each on 1B, 2B, 3D, 4B, and 5A accounting for 12–19.4% of the phenotypic variance (Supplementary Table 5). Five MTAs were detected at position 54.04 cM on chromosome 1A in an environment and BLUP. A stable locus on chromosome 3B for GWS was identified in this study and had a pleiotropic effect on GY in an environment and BLUP. Two important and stable loci for GWS, one on chromosome 5A (AX-95001743 at 12.25 cM) and another on chromosome 6A (AX-94544731 at 6.84 cM) were identified in the present investigation in an environment and BLUP. Ten significant MTAs were detected for the trait GNS on chromosomes 1B (2), 3B (4), and one each on 1A, 2A, 3D, and 5A which explained phenotypic variation of 15.5–24.9%. Marker AX-94494277 (29.49 cM) on chromosome 3D was found consistent for GNS over two environments (E1 and E2) and BLUP in contrast to other markers which were reported in an environment and BLUP. For TGW, 24 MTAs were identified on chromosomes 2A (1), 3A (4), 3B (3), 4A (1), 4B (1), 4D (1), 5A (1), 5D (8), 7A (2), and 7B (2). A stable QTL region on chromosome 5D at 4.8 cM (554.47–555.92 Mb) was associated with TGW in E1 and BLUP. These loci were accountable for 5–15% phenotypic variations for TGW in the present GWAS panel. Marker AX-94732225 (45.96 cM) on chromosome 3B showed maximum positive allelic effect (2.86) and marker AX-94823297 (45.84 cM) on chromosome 3A showed maximum negative allelic effect (–2.42). These two loci explained 7–12 and 8–14% phenotypic variations.

We identified a total of 21 MTAs associated with GY, distributed on chromosome 2B (2), 3D (5), 4B (2), 6A (8), and one each on 1B, 2A, 3A, and 5A (Supplementary Table 5). Two MTAs AX-94784245 (1B) and AX-94407346 (3A) were more significant as these were consistent across two environments and BLUP explaining the phenotypic variation of 22.23–30.51%. A QTL on chromosome 3D was detected for yield spanning an interval of 191.16–203.34 cM. The MTA, AX-94407346 on chromosome 3A was found most promising for GY while showing the highest phenotypic variation of 30.19% across two environments and BLUP. Another important MTA (AX-94761935) associated with yield was observed on chromosome 2B accounting for 30.15% phenotypic variance including BLUP. For HI, 18 MTAs were detected on chromosomes 3A (2), 3B (2), 4B (2), 6A (10), and one each on 1B, and 5B in an environment and BLUP. A stable QTL consisting of 10 MTAs was detected for HI on chromosome 6A (103.98 cM, 535.89–538.05 Mb) showing the phenotypic variance ranging from 29.1 to 40.9% in environment E3 and BLUP. AX-94407346 on chromosome 3A was observed with a maximum phenotypic effect of 41.0% in environment E2 and BLUP for HI. Six MTAs were detected for BM on chromosome 2A (1), 3D (4), and 6A (1) explaining phenotypic variation ranging from 6.0 to 9.0%. The genomic region reported on chromosome 3D covered all four markers and was consistent in two environments and BLUP whereas the other two markers from chromosome 2A and 6A were reported with BLUP only. Furthermore, the allelic effect of these markers ranged between –0.09 and 0.09.

### Spike Fertility-Related Traits

A GWAS was performed for SF-related traits (SF, CW, and SDW) (Supplementary Table 5). For SF, 14 MTAs were identified on chromosomes 3A (3), 3B (3), 4A (2), 5A (1), and 5B (5) in an environment (either E2 or E3) and BLUP explaining 7.1–11.6% of phenotypic variance. For SDW, nine MTAs were detected on chromosomes 1A (2), 3A (3), and one each on 2A, 2B, 3B, and 6A in two environments along with BLUP which explained 8.0–11.0% phenotypic variation. A total of nine MTAs were identified for CW on chromosomes 3A (3), 5B (5) and one on 6B in two or more environments and BLUP explaining 4.2–9.9% phenotypic variation. Three SNPs on chromosome 3A and two SNPs on chromosome 5B were found most stable as detected across three environments and BLUP.

### Pleiotropic Loci

A total of 56 pleiotropic markers were identified, common for highly correlated traits in one or more environments (Supplementary Table 6). Twenty-three loci were found associated with both DH and DA whereas, only one locus AX-94508292 (127.78 cM) on chromosome 7D was found common for DH and DM. Two common SNPs for three phenological traits (DH, DA, and DM) were reported on chromosome 5A at 11.38cM. One marker each on chromosome 1A, 1B, 3B, and 3D showed an association for GWS and GNS. A region between markers AX-94473921 (73.31 cM) and AX-94407346 (74.06 cM) on chromosome 3A was found associated with PH, GY, and HI. Two stable markers *viz*., AX-94544731 on chromosome 6A (6.84 cM), and AX-94475572 on chromosome 2B (102.12 cM) showed a pleiotropic effect on SDW and GWS whereas, another marker AX-94452286 (3B: 83.69 cM) was associated with SDW, GWS, and GNS. Four markers on chromosome 5B anchored at 46.94 cM (AX-94706906 and AX-95632529), 51.91 cM (AX-95131153), and 55.29 cM (AX-94439232) were associated with SF and CW. Marker, AX-94823192 (4A: 45.84 cM) was controlling SF and TGW. Two markers (AX-94823297 and AX-94526152) anchored at 209.17 cM of chromosome 3A were consistently associated with SF, SDW, CW, and TGW. Another marker AX-94842717, anchored to the same position was found associated with DH and DA including SF, CW, and TGW. Likewise, AX-94732225 (3B: 45.96 cM) was found common for SF, GNS, and TGW. One marker on chromosome 1B (AX-94784245), two markers on 4B (AX-94589857 and AX-94461604), and 7 markers anchored at 103.98 cM of chromosome 6A were associated with GY and HI. Three markers from chromosome 3D one at 194.61cM (AX-94598770) and two at 203.07cM (AX-94493158 and AX-94464974) were consistently associated with GY and BM.

### Genotype-Phenotype Network

**FIGURE 4:**
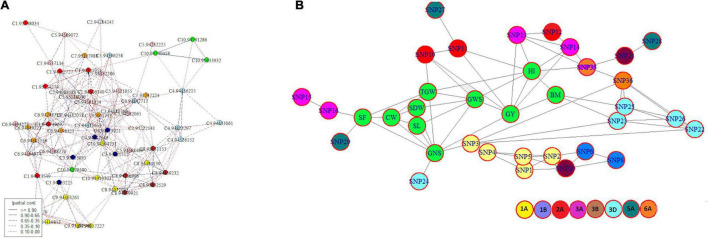
**(A)** Intra- and inter-chromosomal conditional interaction network among 77 significant SNP markers across bread wheat genome where each colour represents a different chromosome and **(B)** a genotype-phenotype network showing interaction among traits and associated SNPs where traits are highlighted in green and other colours designates different chromosomes.

The ‘*netgwas*’ efficiently estimate pairwise interactions between different loci in a genome while adjusting for the effect of other loci. Network analyses due to the conditional dependence feature reduce the number of possible SNPs and provide an interaction network of key SNPs associated with studied traits. Development of the geno-pheno network (Figure 4A) with 77 key SNPs, associated with 10 traits (SF, SL, CW, SDW, TGW, GNS, GWS, GY, BM, and HI) indicated the inter- and intra- chromosomal genetic control of these traits. Inter-chromosomal connections were identified between 26 SNPs belonging to chromosomes 1A, 1B, 2A, 3A, 3B, 3D, 5A, and 6A (Supplementary Table 8) indicating the importance of these chromosomes in the phenotypic variation of the studied traits. Here, we identified 11 key SNPs (SNP 3, SNP 10, SNP 11, SNP 13, SNP 14, SNP 16, SNP 23, SNP 24, SNP 25, SNP 29, SNP 35) distributed across 5 chromosomes (1A, 2A, 3A, 3D, and 6A) interacting with multiple traits (Figure 4B). The geno-pheno network describes the complex genetics of phenotypic correlations between studied traits. Furthermore, these SNPs can be used as a minimal marker system for the simultaneous improvement of the studied traits.

### Favourable Alleles

Increasing numbers of favourable alleles showed a significant effect on the increase in phenotypic values of SF, GNS, GWS, SDW, TGW, HI, and GY whereas, a decrease in CW and PH (Supplementary Figure 2). In the above-mentioned traits, the number of favourable alleles ranged between 5 (SDW) and 16 (PH). The *r*^2^ further ranged between 0.02 (CW) and 0.22 (GNS). These results indicated that the increase in GY depends on the increasing number of favourable alleles associated with GY and other correlated traits. The genotype WH1080 carried the highest 12 favourable alleles for GY. Likewise, for SF maximum of 12 favourable alleles were carried by HD3086, MACS6222, MP1201, MP3211, and VL738, each. Furthermore, genotype PBW373 contained the maximum number (63) of favourable alleles for the traits PH, GY, HI, GNS, GWS, TGW, SDW, and CW whereas, L25AMB carried the least numbers (8) of favourable alleles for traits SDW and CW. The genotype with the highest overall grain yield of 117.56 g, PBW396 carried 37 favourable alleles for the traits PH, GY, HI, GNS, GWS, TGW, and SDW. Thus, these genotypes can prove as efficient sources of favourable alleles for improving the desirable traits.

### Candidate Gene Prediction and Homology Modeling

In total, 102 SNPs were physically mapped to IWGSC *RefSeq v1.1* with high confidence. To identify the putative candidate genes, 1.5 kb upstream and downstream regions of the mapped SNPs were annotated using EnsemblPlant biomart. It led to the identification of 81 putative candidate genes. Among these, 69 were overlapping, and 11 were within 1.5 kb window (Supplementary Table 5). Furthermore, *in silico* expression analysis was carried out using a publically available RNA-seq expression database of polyploid wheat (see footnote 3). Of 81 putative candidate genes, 61 showed growth stage-specific differential expression reported in the Chinese Spring cultivar’s spikes, grains, stem, and leaf tissues (Supplementary Table 7). The range FPKM value was between 0.68 (*TraesCS3B02G105100*) and 301.43 (*TraesCS1B02G380800*). Detection of the underlying genes related to the trait provides further reliability of the identified MTAs.

Based on the literature survey and to the best of our knowledge, 22 novel SNPs were identified in the present investigation (Supplementary Table 9) that were associated with GY, GNS, GWS, SDW, SF, TGW, and CW. However, 18 of these SNPs physically mapped to IWGSC *RefSeqv1.1* with high confidence; 11 SNPs overlapped by candidate genes for which no reliable GO term was found in the database. Therefore, protein modeling was carried out with the translated amino acid sequences. Excellent 3D structures of translated proteins were projected using a template searched by PSI-BLAST (Supplementary Figure 3 and Table 1). For gene *TraesCS5D02G545100* no suitable template was obtained within our cutoff value of identity percentage (≥25%). Identity between query sequences and their respective templates ranged from 26.26 to 47.94% whereas, GMEQ ranged between 0.27 and 0.6. A range of 349.03–1978.18 and 0.26–1.72 Å was reported for FATCAT and root to mean square deviation (RMSD) respectively. Furthermore, values from ERRAT and Verify3D ranged from 58.93 to 95.83% and 64.18 to 95.22%, respectively (Supplementary Table 9). Ramachandran plots indicated that among the predicted 3D models 81–95% amino acids were in favoured regions.

**TABLE 1:** A list of predicted proteins translated by the IWGSC genes overlapping 10 novel SNPs.

SNP	Trait	Gene stable ID	Predicted protein	Function	References
AX-94452286	GNS, GWS, SDW	TraesCS3B02G104700	Xylan *O*-acetyltransferase	Polysaccharide acetylation and improved water use efficiency	Shang et al., 2020
AX-94493158	GY	TraesCS3D02G538400	Pectinacetylesterase/NOTUM	Catalyses the deacetylation of pectin, cell elongation, pollen formation	Gou et al., 2012; Kakugawa et al., 2015
AX-94464974	GY	TraesCS3D02G538500			
AX-94747224	TGW	TraesCS5A02G428800	Coat protein complex II (COPII) of type Sec23a/24a c	Integrity of cell organelles	Wang et al., 2016
AX-95632529	CW, SF	TraesCS5B02G104300	Dipeptidyl Aminopeptidase IV	Remobilisation and utilisation of storage proteins	Simpson, 2001
AX-94484139	SF	TraesCS5B02G156900	MFAP1 and Snu23 complex	Contact with Prp38 via ER/K motif-stabilizers single α helices	Ulrich et al., 2016
AX-95097548	TGW	TraesCS5D02G547200	Recognition of *Peronospora Parasitica* 13	Host pathogen interaction	Bittner-Eddy et al., 2000
AX-94542611	TGW	TraesCS5D02G548200			
AX-94610590	TGW	TraesCS5D02G548300			
AX-94389673	TGW	TraesCS7A02G512300	Indole-3-glycerol phosphate synthase	Indole acetic acid (IAA) biosynthesis	Ouyang et al., 2000

## Discussion

### Adaptation Related Traits

Considering the diversity of agro-climatic zones in India, DH plays a major role in the wider adaptability of the wheat genotypes. Earlier findings reported that genes associated with flowering are mainly located on chromosomes 1A, 2B, 3A, 3B, 5A, 6A, 6B, 7A, 7B, and 7D (Kobayashi et al., 2016; Lozada et al., 2017; Ogbonnaya et al., 2017; Hassouni et al., 2019; Sheoran et al., 2019). In the present study, two stable clusters associated with DH, DA, and DM were identified on chromosome 3A at locus 127.08–127.87 cM and the other at 209.17 cM across all the studied environments. However, on chromosome 5A, we obtained two highly stable regions associated with phenological traits (DH, DA, and DM) one at locus 11.38 cM (586.60–588.37 Mb) that may be marked within the boundaries of gene *TraesCS5A02G391400* and *TraesCS5A02G392000* and the second, at an interval of 89.02–92.18 cM (533.27–546.30 Mb) which can be marked in the limits of three genes, namely *TraesCS5A02G320100*, *TraesCS5A02G320300*, and *TraesCS5A02G392000.* Pleiotropic SNP AX-94796479 of the first region (588.37 Mb) on chromosome 5A identified within gene *TraesCS5A02G392000* at 3′ UTR encodes COBRA-like protein. This protein is involved in the cellulose deposition in mucilage secretory cells in *Arabidopsis* (Ben-Tov et al., 2015). The region (586.60-588.37Mb) identified in this study, overlapped with the vernalisation gene *Vrn-A1* (587.4 Mb) and *TaAGLG-5A* gene (588.0 Mb) on chromosome 5A, the core regulators in the vernalisation pathway which regulates plant development (Yan et al., 2003; Wang et al., 2017). Another promising region on chromosome 6A in an interval of 33.79–47.56 cM was detected associated with DH and DA.

**FIGURE 5:**
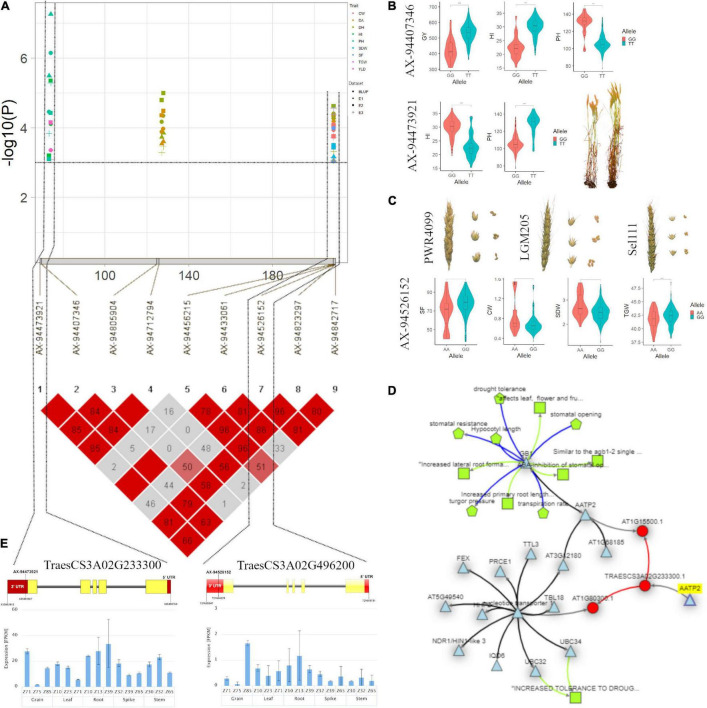
**(A)** Local Manhattan plot and linkage disequilibrium (LD) heatmap of stable SNPs associated with grain yield (GY), harvest index (HI), plant height (PH), spike fertility (SF), chaff weight (CW), spike dry weight (SDW), and thousand-grain weight (TGW) on chromosome 3A. **(B)** Effect of different alleles of associated SNP markers on the phenotypic values of GY, HI, and PH. **(C)** Effect of different alleles of marker AX-94526152 on phenotypic values of SF, SDW, CW, and TGW. **(D)** Network generated for gene *TraesCS3A02G233300* (AATP2). **(E)** Structure of two candidate genes and their *in silico* tissue and growth specific expression profile.

In the case of PH, significant MTAs were identified on chromosomes 1A, 2B, 3A, 3B, and 6A. The marker AX-95099974 mapped at 104.59cM on chromosome 2B is in close proximity to *Rht4* (Ellis et al., 2005; Sheoran et al., 2019). On chromosome 1A, we detected a stable genomic region for PH spanning in an interval of 74.11–74.86 cM (320.22–439.07 Mb) within two genes is likely to be a new region for the trait. Another stable region for PH was identified on chromosome 3A spanning the region between 73.31 and 74.06 cM (435.80–457.79 Mb) within *TraesCS3A02G233300* gene annotating ADP, ATP carrier protein (Figures 5A,B). This protein is responsible for the lower shoot weight and less tolerant to high light conditions in *Arabidopsis* (Yin et al., 2010). The gene, *TraesCS3A02G233300* further showed the significant (*p* < 0.05) regulatory association (Figure 5D) with phenotypes that closely affect the PH and HI such as sensitivity to growth inhibitors, primary and lateral root development, turgor pressure, and leaf, flower, and fruit development, in Knetminer network that further validates the results (Figure 5C,E).

### Spike Fertility and Related Traits

Hotspots for SF were identified on chromosomes 3A, 3B, 4A, 5A, and 5B harbouring genomic regions with multiple traits. A potential genomic region for SF was identified at 209.17 cM (721.66 Mb) on chromosome 3A within gene *TraesCS3A02G496200* co-located with additional three traits, namely, CW, SDW, and TGW. This region aligned with the reported region (714.4–725.8 Mb) on chromosome 3A by Li et al. (2019), significantly associated with GY, kernel number per spike (KNS), kernel width (KW), SDW, PH, uppermost internode length (UIL), and flag leaf length (FLL) showing a significant effect on GY. It is also co-localised with thousand kernel weight (TKW) QTL, namely *QGw.nau-3A* (720.59 Mb) and *QTgw-3A1* (721.22 Mb) (Jia et al., 2013; Liu et al., 2014) and AX-108992368 (721.32 Mb) for GNS (Li et al., 2019). Here, it is noteworthy that the discovery of a stable genomic region for SF with a significant pleiotropic effect on SDW, CW, and TGW might be valuable for breeding purposes. Recently, Pretini et al., 2020 also identified and validated a promising QTL (*QFE.perg-3A*, 51.6 cM, 685.12 Mb) associated with FEm (Fruiting efficiency at maturity) on chromosome 3A which is about 36.55 Mb apart from SNP reported in this study. Therefore, this region could be a promising breeding target for genetic improvement of sink strength. PC biplot analysis also supported these findings which showed clustering of SF with SDW, CW, and TGW. We detected a co-localised locus AX-94732225 (45.96 cM, 29.61 Mb) on chromosome 3B for SF, GNS, and TGW within gene *TraesCS3B02G056100* near the same position as a FEh haplotype/SNP reported by Basile et al. (2019).

Furthermore, a stable genomic region for SF was observed on chromosome 4A (45.84 cM, 29.07 Mb) within gene *TraesCS4A02G036600*. On chromosome 4A, we identified an MTA AX-94582600 at locus 66.89 cM (41.91 Mb) that was not considered significant as it was detected in only one environment. It was found within the gene *TraesCS4A02G050800* encoding Gibberellin_3-beta-dioxygenase_4 plays a pivotal role in controlling growth and development especially known for its importance in spikelet fertility of crops (Kwon and Paek, 2016; Alqudah et al., 2020). Another MTA (AX-94950716) at locus 210.24 cM (726.44 Mb) was detected in environment E2 and BLUP within gene *TraesCS4A02G462300* which was located near the reported position by Pradhan et al. (2019) annotating Haloacid dehalogenase-like hydrolase domain-containing protein Sgpp which enhanced phosphatase activity and biomass in rice (Pandey et al., 2017). It is noteworthy, that these genes were also strongly expressed in the grain (Grain_Z71, FPKM-49.49) and spike (Spike_Z39, FPKM-13.61) revealing the importance of these regions which could further be dissected to prove their role in trait improvement. Comparing with the previously reported region for SF on chromosomes 1A, 2A, 3B, 4A, 5A, 6A, 6B, 7A, and 7D (Basile et al., 2019; Pradhan et al., 2019; Pretini et al., 2020), we discovered five MTAs for SF co-located with CW on chromosome 5B which might be considered as a novel region indicating the significance of assimilate distribution in CW in the improvement of SF.

### Yield and Component Traits

In the present study, a significant genomic region associated with GY, SL, and HI has been identified on chromosome 1B at loci 8.24 cM, which is in proximity to yield QTL (*QYld.aww-1B.1*) from RAC875/Kukri and QTL for yield components and relative leaf expansion rate from Drysdale/Gladius^8^. This validates the stability of the genomic region for use as potential QTLs for marker-assisted selection aiming high yielding wheat lines. In previous studies, chromosome 3A is known to be a hotspot that contains valuable QTLs for GY and yield-related traits in wheat^9^ (Mengistu et al., 2012; Rustgi et al., 2013). Many cloned yield and component-related genes *viz*., *TaTAR2.1-3A, TaGS5-A1/3A, TaTGW6-*A1 are mapped on chromosome 3A (Wang et al., 2015; Hanif et al., 2016; Ma et al., 2016; Shao et al., 2017). The present study also reports important clusters on chromosome 3A, most stable on position 457.79 Mb (SNP AX-94407346, 74.06 cM) for GY observed in two environments and BLUP. However, this region is 75.64 Mb away from the gene *Ta-TGW6-A1* associated with TGW (Hanif et al., 2016). This SNP also had a pleiotropic effect with PH and HI, thus could be considered as a potential genomic region for future functional validation studies. Recently, Martinez et al. (2021) also reported that PH, DH, and GY are interrelated traits in wheat. Another potential genomic region identified in this study for GY was on chromosome 5A at locus AX-94472479 located at 417.88 Mb (72.2 cM). This is within the intron variant of the gene *TraesCS5A02G207000* which falls within the confidence interval of *Q.Gnu.uwa-5A-1* detected in the Synthetic W7984 x Opata M85 population by Onyemaobi et al. (2018). This gene encodes for the Transcription Initiation factor TFIID subunit 2. This protein along with *POW1 (put on weight 1*) is involved in the functioning of grain size regulation by repressing the transcription activity of the interacting protein TAF2, a highly conserved member of the transcription initiation complex TFIID in rice (Zhang et al., 2019). Two potential genomic regions were identified in this study on chromosome 3D, one at 29.49 cM governing GWS and GNS and the other at locus 203.07 cM (611.14–611.16 Mb) associated with GY and BM within two genes *TraesCS3D02G538400* and *TraesCS3D02G538500* respectively are likely to be new. Moreover, *TraesCS3D02G538400* showed high expression in spike (FPKM-9.9 at Zadok stage 39) and both the genes encode a protein pectin acetylesterase (PAE) which has an important role in plant tissue development (reduce inflorescence, stem height) in *Arabidopsis* (de Souza et al., 2014). Thus, it can be hypothesised that in the present study these two PAE genes (*TraesCS3D02G538500* and *TraesCS3D02G538400*) might have affected GY by affecting SF, GNS, and TGW.

**FIGURE 6:**
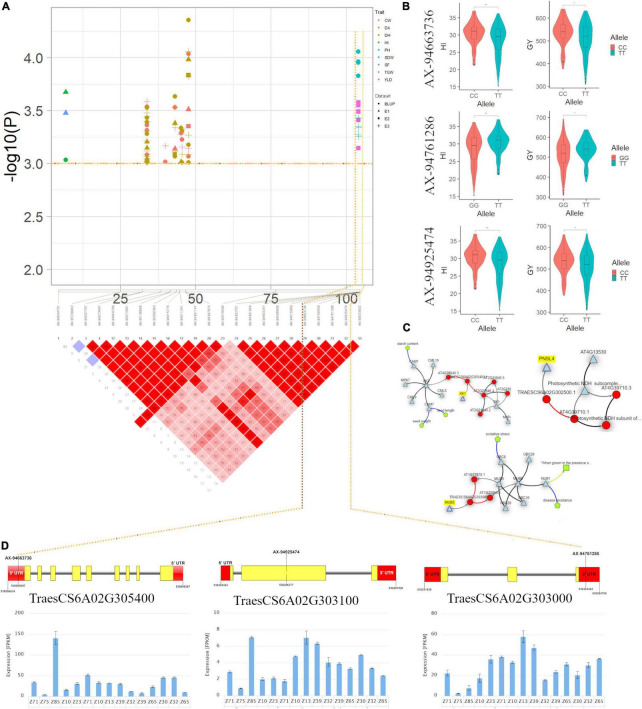
**(A)** Local Manhattan plot and linkage disequilibrium (LD) heatmap of stable SNPs associated with grain yield (GY) and harvest index (HI) on chromosome 6A. **(B)** Effect of different alleles of associated SNP markers on the phenotypic values of GY and HI. **(C)** Network generated for three candidate genes. **(D)** Structure of three candidate genes and their *in silico* tissue and growth specific expression profile.

On the genetic map, a genomic region at 103.98 cM (536.25–538.05 Mb) on chromosome 6A associated with GY and HI appeared to be another important region in the current study (Supplementary Table 6 and Figures 6A–D). This region harboured three candidate genes *viz*., the first gene *TraesCS6A02G303000* encodes a membrane-anchored ubiquitin-fold protein, second candidate gene, *TraesCS6A02G303100*, located at 536.25 Mb encodes a protein tRNA [(carboxymethyl uridine(34)-5-O)-methyltransferase] which plays a role in stress-response and a third candidate gene *TraesCS6A02G302500* associated with GY and HI annotates a protein Peptidylprolyl *cis-trans* isomerase (PPIases). PPIase is reported essential for stabilisation of photosystem II and their upegulation leads to a higher photosynthesis rate in wheat (Wang et al., 2014). In an earlier report, Lee et al. (2014) identified a QTL *QTKW-6A.1* in the same region for TGW indicating that this should be the potential novel locus for determining GY and its component. Furthermore, the Knetminer network (Figure 6C) revealed that homologues of these genes in *Arabidopsis* have a regulatory association with the similar traits for which these genes have been found associated in this study. For instance, the gene *Traes6A02G305400* that overlapped the SNP AX-94663736 associated with GY and HI showed a regulatory association with traits seed length and seed weight, at *p* < 0.05, which are the key factors for deciding GY and HI in any cereal crop.

Spike length plays a significant role in improving wheat yield (Guo et al., 2017). Earlier studies have reported stable QTLs/MTAs for SL on chromosomes 1A, 1B, 3A, 3B, 4A, 4B, 4D, 5A, 5B, 6A, 6B, 6D, and 7A (Liu et al., 2018; Hu et al., 2020; Pretini et al., 2020). The three stable MTAs associated with SL, one on chromosome 1D at locus 95.7 cM within *TraesCS1D02G147600* and the other two at locus 209.9cM (634.27 Mb) on chromosome 5B have not been reported earlier and are potentially novel MTAs responsible for SL (Supplementary Table 5).

For TGW, we observed a stable MTA AX-94747224 (13.4, 613.47 Mb) on chromosome 5A within gene *TraesCS5A02G428800* located 32.2 Mb away from the reported gene *TaNAC2-5A* associated with yield (He et al., 2015) and 8.06 Mb distal from *QTKW.ndsu.5A.2* for TGW (Kumar et al., 2016) indicating this region is highly significant for TGW and has the potential for improving GY. We detected a stable genomic region for TGW on chromosome 5D harbouring seven MTAs spanning an interval of 554.47–555.92 Mb. This region is very near to the candidate gene *TaCWI-5D* (557.9 Mb) for TGW (Jiang et al., 2015). Notably, the MTA AX-94389673 within gene *TraesCS7A02G512300* on chromosome 7A (163.4 cM, 699.74 Mb) associated with TGW lies in close vicinity to the gene *TaAPO-A1* (673.1–868.2 Mb) and overlapped with the QTL *QTKW-7AL-AN* for TGW reported by Quarrie et al. (2005) and Muqaddasi et al. (2019), respectively, which reveals the importance of this genomic region in wheat improvement. Similarly, in the present study, significant MTA for TGW on chromosome 7B was detected at locus 643.29 Mb (96.68 cM) within the gene *TraesCS7B02G378700* which is only 4.0 Mb proximal to QTL *qSn-7B.2* for spike number (Fan et al., 2019) and 42.2 Mb distal to QTL *QTKW.caas-7BL* for TGW (Gao et al., 2015).

For GNS, 10 significant MTAs distributed on chromosomes 1A, 1B, 2A, 3B, 3D, and 5A were found consistent in two environments and BLUP. A genomic region identified on chromosome 1B at 555.29 Mb (35.34 cM) was almost at the same position as QTL *QKNS.caas-1BL.2* reported by Li et al. (2018). A GNS QTL (*KNS-gwm312)* mapped on chromosome 2A reported by Wang et al. (2012) is near to the position of MTA AX-94463225 within the synonymous variant of gene *TraesCS2A02G563400*. This locus is close to the gene *TaFlo2-A1* (23.73 Mb) (Sajjad et al., 2017) associated with TGW showing the causal effect of the gene. It is also noticeable that an important genomic region at 83.69 cM (70.27–71.14 Mb) within gene *TraesCS3B02G104700* co-localised for SDW, GNS, and GWS on chromosome 3B is likely to be novel. Literature survey suggests that another consistent loci identified in the study for GNS and GWS on chromosome 3D at 29.49 cM across environments and BLUP have no previous reference. Therefore, these two regions on chromosomes 3B and 3D represent two novel loci governing GNS and GWS in wheat.

Little information is available in various databases on the functionality of candidate genes associated with novel SNPs identified in the present GWAS. Therefore, homology modeling was opted to identify proteins translated by these candidate genes and their role in the expression of associated traits. For this purpose, we searched for a homologous template across the NCBI database and selected the top three hits for modeling purposes. Templates that showed high similarity with our protein sequences belonged to *A. thaliana*, *Homo sapiens*, *Chaetomium thermophilum* (a thermophilic filamentous fungus), *Stenotrophomonas maltophilia* (an aerobic, non-fermentable, Gram-negative bacterium), and *Staphylococcus aureus* (a Gram-positive, round-shaped bacterium). Values of FATCAT and RMSD indicated significant similarity between query sequences and corresponding PDB templates at *p* < 0.05. The accuracy of predicted models was evaluated on the basis of ERRAT, Verify3D, and Ramachandran plot. In all the predicted structures we reported that >80% of amino acids were in favoured regions of the Ramachandran plot which is an acceptable range for an accurate model (Gautam et al., 2019). However, for gene *TraesCS5D02G545100* values of ERRAT and 3D were not in the acceptable range hence the structure of this gene was not considered accurate. Thus, we rebuilt accurate models for the rest ten IWGSC high confidence genes and submitted them to the protein model database (PMDB). Three genes *TraesCS5D02G548200*, *TraesCS5D02G548300* and *TraesCS5D02G547200* were translated into Recognition of *Peronospora parasitica* 13 (RPP-13)- like gene. This protein has been found to be crucial for host-pathogen interaction in various plant diseases and subjects to defence mechanisms mainly in the case of downy mildew (Bittner-Eddy et al., 2000). Bouchabke-Coussa et al. (2008) demonstrated the association between ESKIMO-1 protein and improved water use efficiency (WUE) speculating that GY can be improved through allele selection or manipulation of the ESKIMO-1 gene. In our study, SNP AX-94452286 producing a significant association with three traits GWS, GNS, and SDW was reported in a gene (*TraesCS3B02G104700*) encoding ESKIMO-1 protein. All of these traits are highly correlated with WUE (Shang et al., 2020). Trait TGW was found to be associated with SNP AX-94747224 of gene *TraesCS5A02G428800* encoding Coat protein complex II (COPII) of type Sec23a/24a complexed with SNARE. COPII proteins are crucial in maintaining the integrity of the Golgi complex and endoplasmic reticulum, and vacuolar transportation of storage proteins. Storage proteins play an important role in the development of grain and its final weight. Mutation in the COPII type gene has been found responsible for reduced TGW in rice due to defects in vacuolar protein (Wang et al., 2016). An SNP AX-94389673 affecting the TGW in our study was found in a gene *TraesCS7A02G512300*. This gene translates into Indole-3-glycerol phosphate synthase (IGPS) which is a key enzyme in the pathway of indole acetic acid (IAA) biosynthesis (Ouyang et al., 2000) and plays an important role in determining grain weight by affecting grain size (Nadolska-Orczyk et al., 2017). Two genes (*TraesCS3D02G538500* and *TraesCS3D02G538400*) encoding Notum protein are found to be associated with GY. Instead, it is well-known that notum deacylates Wnts to suppress signalling activities (Kakugawa et al., 2015). Wnt is a family of highly conserved signalling proteins regulating various developmental processes. The existence of Wnt protein-mediated signalling in plants is still less explored. These two genes further showed more than 65% similarity with Pectinacetylesterase/NOTUM (PAE/NOTUM: IPR004963) genes of *A. thaliana* (AT3G09405 and AT4G19420) and *Oryza sativa indica* (BGIOSGA000013 and BGIOSGA003380) in EnsmblePlant PBLAST search. PAE catalyses the deacetylation of pectin which is a key component of the primary cell wall in plants. Previously, Gou et al. (2012) demonstrated reduced cell elongation, pollen formation, and increased sterility due to overexpression of the PAE gene in tobacco. Thus, it can be hypothesised that in the present study these two PAE genes (*TraesCS3D02G538500* and *TraesCS3D02G538400*) might have affected GY by means of affecting SF, GNS, and TGW.

Thus, we identified 22 novel loci in the present GWAS that produce 32 MTAs, 11 of which overlapped by high-confidence IWGSC genes (Supplementary Table 9). Furthermore, with the help of a stringent modelling framework of ‘*netgwas*,’ which provides a discrete and complex graphical network, we studied the complex interaction between the: (1) significant SNPs, (2) phenotypes, and (3) SNPs and phenotype. As a result, the ‘*netgwas*’ empowered us to narrow down the number of significant markers to the eleven most promising SNPs (Supplementary Table 8) for the simultaneous improvement of SF, GY, and closely related traits. Previously, Alqudah et al. (2020) also adopted a similar strategy to identify the most promising SNPs for the simultaneous improvement in SF and associated traits. However, the information available on various databases was insufficient to confirm the functionality of these genes. In this situation, the homology modeling of these genes proved to be a potential tool not only for validation of the function of these genes but also for identifying their importance in future wheat improvement programs.

## Conclusion

Spike fertility and GY are closely associated therefore, improving spikelet fertility can be a possible way of improving the yield potential of a genotype. Although these traits are normally sensitive to the environment and show high GxE interaction, their considerable heritability across the environments in this study indicates possibilities of their exploitation toward improving grain yield. Based on the GWAS result, 255 MTAs identified for 15 traits were further narrowed down to 11 key MTAs using the geno-pheno network. In total, 22 novel MTAs were detected that have been validated with gene expression analysis and homology modelling. MTAs found in the study with the corresponding favourable allele shall be converted into breeder friendly marker system such as KASP (Kompetitive Allele-Specific PCR). A panel of the selected KASP markers shall be utilised to screen the crossing block genotypes. This will not only help in prioritizing the identified genotypes in the crossing program but also for early generation screening of the segregating lines. These loci will add on precision in future breeding programs through marker-assisted selection. Additionally, functional annotation of the genomic region within the 1.5 Kb window of each identified SNP allows us to recognize candidate genes. Upstream analysis of these genes will help to improve the understanding of key regulatory networks and the underlying mechanism of the studied traits.

## Data Availability Statement

The original contributions presented in the study are included in the article/Supplementary Material, further inquiries can be directed to the corresponding author/s.

## Author Contributions

RT, AR, and DK conceived the theme of study. SSh, SJ, MI, UA, JJ, and GT analysed the data. SSh, SJ, MI, PS, RSi, and RT drafted the manuscript. NR, RSh, GS, Sabhyata, and AGa conducted the phenotyping. AGu was instrumental in arranging the genotypes for study. RT, GS, AR, GPS, and DK provided overall guidance and edited the manuscript. All the authors read and approved the final version of the manuscript.

## Conflict of Interest

The authors declare that the research was conducted in the absence of any commercial or financial relationships that could be construed as a potential conflict of interest.

## Publisher’s Note

All claims expressed in this article are solely those of the authors and do not necessarily represent those of their affiliated organizations, or those of the publisher, the editors and the reviewers. Any product that may be evaluated in this article, or claim that may be made by its manufacturer, is not guaranteed or endorsed by the publisher.

## Footnotes

1
http://www.sas.com

2
https://www.R-project.org/

3
http://plants.ensembl.org/Triticum_aestivum/

4
https://urgi.versailles.inra.fr/blast_iwgsc/blast.php

5
https://wheat.pw.usda.gov/WheatExp/

6
http://www.rcsb.org/pdb/home/home.do

7
https://swissmodel.expasy.org/

8
http://hdl.handle.net/2440/127107

9
https://archive.gramene.org/db/qtl

## Supplementary Material

Supplementary Table 1 Details of 200 genotypes used in the present study.

Supplementary Table 2 Analysis of phenotypic data of traits studied in a diverse panel.

Supplementary Table 3 A pooled ANOVA.

Supplementary Table 4 Basic statistical analysis of SNP markers used for genome-wide association study (GWAS) of 200 wheat genotypes.

Supplementary Table 5 List of significant marker-trait associations (MTAs) reported in present genome-wide association study (GWAS) for 15 traits.

Supplementary Table 6 List of single nucleotide polymorphisms (SNP) markers showing pleiotropism.

Supplementary Table 7 Functional annotation of candidate genes.

Supplementary Table 8 List of SNPs used for developing genotype-phenotype network using ‘netgwas.’

Supplementary Table 9 List of 22 novel SNPs reported in present GWAS for SF, GY and their associated traits.

Supplementary Figure 1 Quantile–quantile (Q-Q) scale representing expected vs. observed *–log_10_P* value for all 15 metric traits.

Supplementary Figure 2 Linear regression between number of favourable alleles and BLUP values observed for selected traits.

Supplementary Figure 3 Predicted 3D structures of proteins translated by candidate genes.
