# Supplementary figures and images for: Genome-Wide Association Study and Post-genome-Wide Association Study Analysis for Spike Fertility and Yield Related Traits in Bread Wheat
Source: Front Plant Sci. 2022 Feb 11;12:820761. doi: 10.3389/fpls.2021.820761 (PMC8873084; doi:10.3389/fpls.2021.820761)

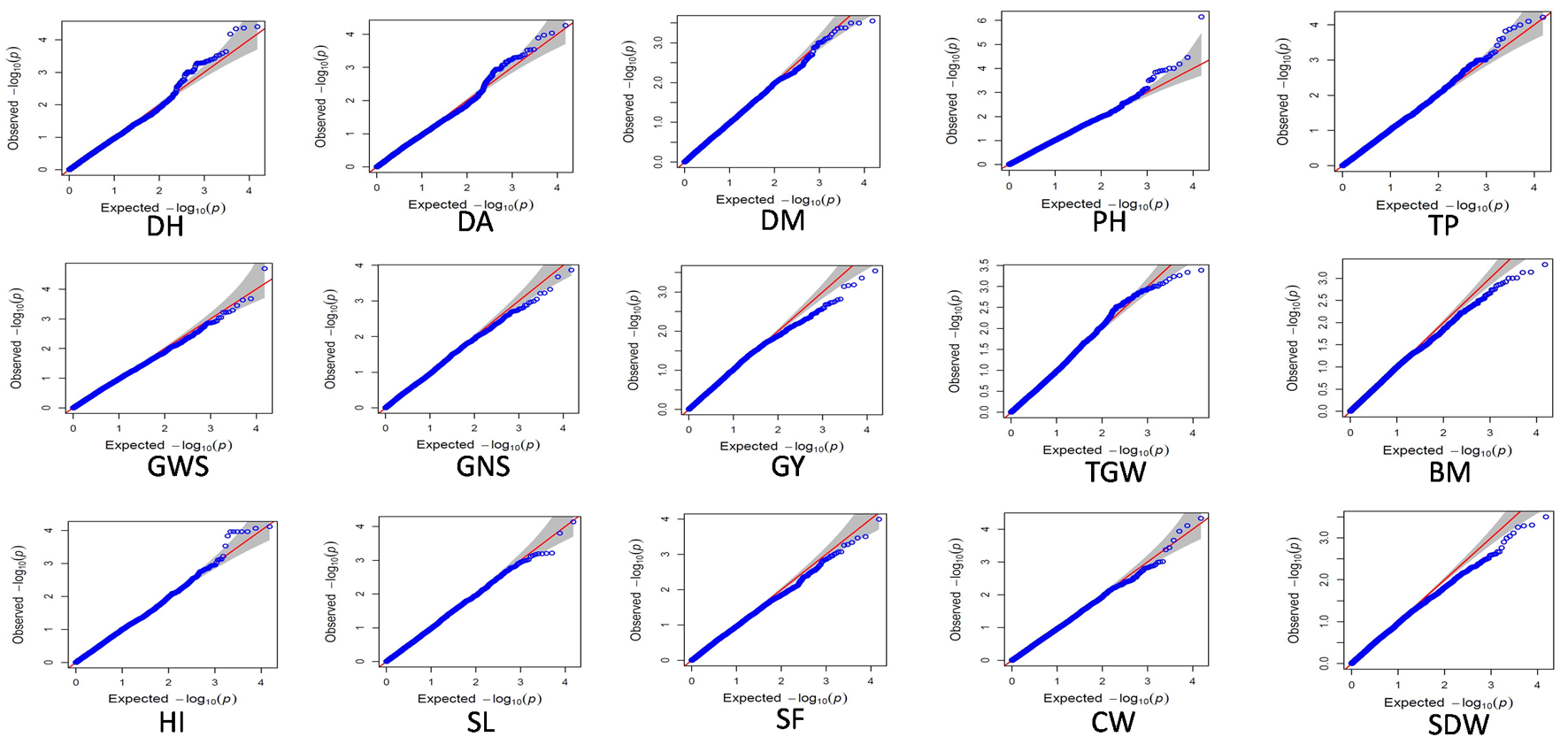

Supplement: Supplementary Figure 1 — Quantile–quantile (Q-Q) scale representing expected vs. observed –log10P value for all 15 metric traits. [file Image_1.TIF]

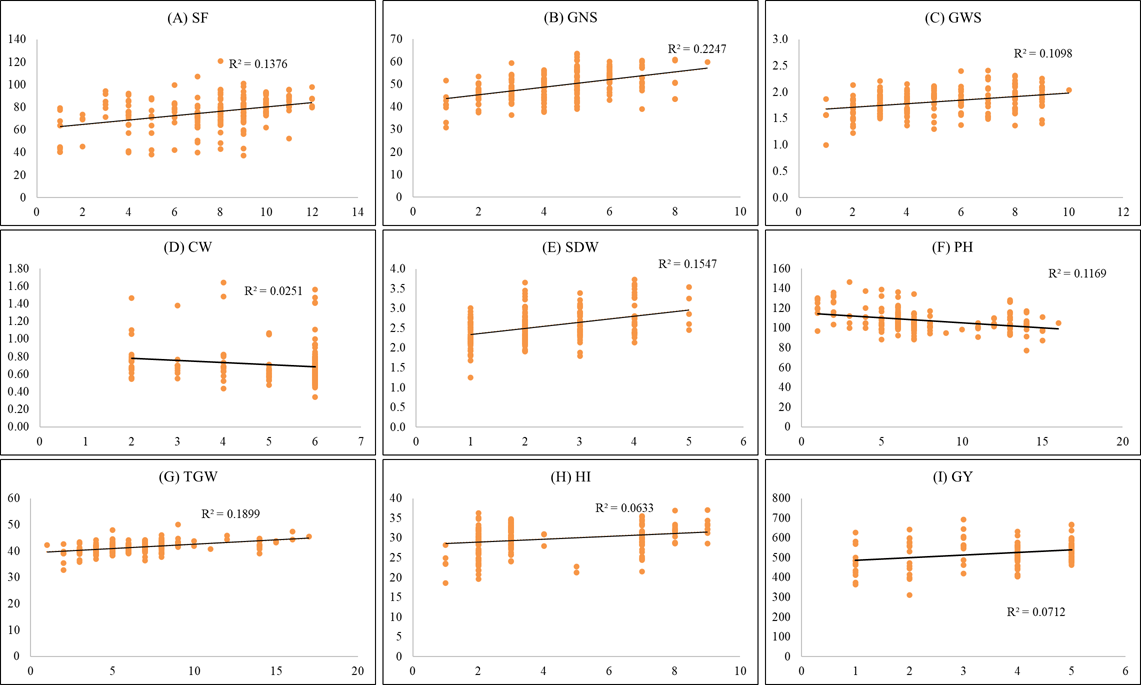

Supplement: Supplementary Figure 2 — Linear regression between number of favourable alleles and BLUP values observed for selected traits. [file Image_2.TIFF]

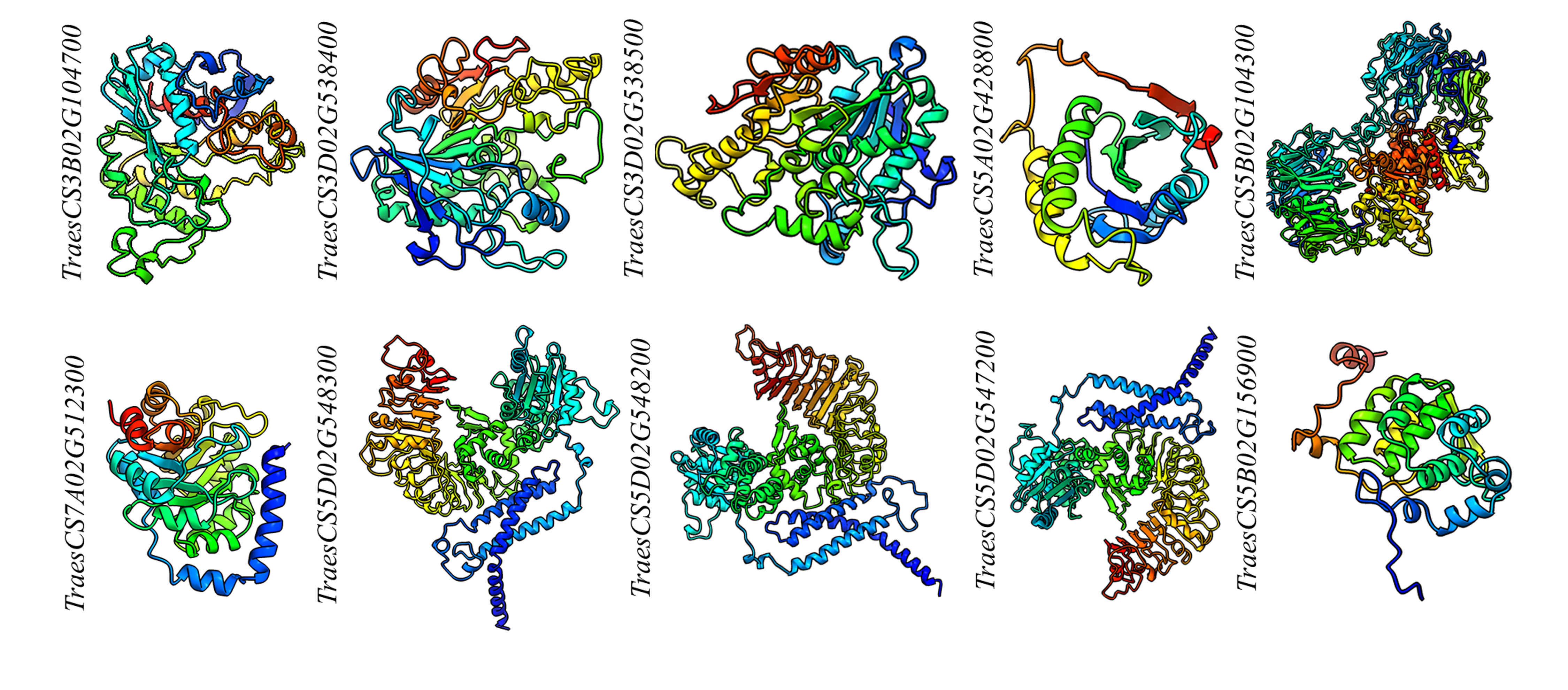

Supplement: Supplementary Figure 3 — Predicted 3D structures of proteins translated by candidate genes. [file Image_3.TIFF]
